# Traumatic events: exploring associations with maternal depression, infant bonding, and oxytocin in Latina mothers

**DOI:** 10.1186/s12905-018-0520-5

**Published:** 2018-02-01

**Authors:** Sandraluz Lara-Cinisomo, Kefu Zhu, Kexin Fei, Yumeng Bu, Alexandria P. Weston, Uma Ravat

**Affiliations:** University of Illinois at Urbana-Champaign, Champaign, IL USA

**Keywords:** Trauma, Abuse, Postpartum, Mothers, Infant bonding, Oxytocin, Depression

## Abstract

**Background:**

Childhood and adulthood traumatic experiences negatively impact maternal-infant bonding and increase risk of postpartum depression (PPD). Lower oxytocin levels have also been associated with PPD and compromised mother-infant bonding. Despite advances in these areas of investigation, much of the research has not included Latinas, who are important because they have high rates of fertility, traumatic events, and PPD.

**Methods:**

To address gaps identified in the literature, we explored associations between traumatic life events, PPD, and bonding subscale scores (e.g., Impaired Bonding, Rejection and Anger, Anxiety about Care) in a sample of 28 Latinas. We also examined associations between these factors and oxytocin (OT). Wilcoxon signed-rank tests were employed to examine differences in subscale scores over time. Kruskal–Wallis one-way analysis of variance was used to examine differences in bonding subscale scores and OT by maternal depression status and traumatic events. We also explored interaction effects of traumatic events and OT AUC on bonding subscale scores.

**Results:**

Women with PPD at 8 weeks had significantly higher Rejection and Anger subscale scores (*p* = 0.054) than non-PPD women, where higher scores represent more compromised bonding. Significant differences in Rejection and Anger (*p* = 0.042) and Anxiety about Care (*p* = 0.005) by adulthood traumatic histories were observed at 8 weeks postpartum. There was also a significant difference in Anxiety about Care scores at 4 weeks postpartum (*p* = 0.024) and Impaired Bonding at 8 weeks postpartum (*p* = 0.041) by trauma events involving an infant. There was a significant interaction between OT and childhood sexual abuse on Impaired Bonding (*p* = 0.038).

**Conclusion:**

We observed differential responses in bonding subscale scores by traumatic histories. Women who experienced a trauma involving an infant had higher compromised bonding scores, whereas those with adulthood traumatic histories, such as intimate partner violence, had lower scores. We also found an interaction between childhood trauma and oxytocin levels on bonding scores, suggesting a physiological response to early abuse that can have implications on mothers’ bonding perceptions. These preliminary results suggest the need for additional research on the long-term emotional and physiological effects of traumatic events occurring prior to parturition.

## Background

Mother-infant bonding is important because it represents maternal feelings about her infant as well as her emotional connection to the infant [1]. While attachment and bonding are often used interchangeably, bonding captures the mother’s process rather than the mother-infant relationship or dyadic interactions. Prior maternal experiences, such as traumatic experience in childhood and adulthood, have been shown to negatively impact maternal-infant bonding [2]. Muzik et al. found that a sample of 97 African American and White mothers with childhood histories of neglect exhibited significant bonding impairment at 6 weeks, 4 months, and 6 months postpartum compared to healthy controls [1]. Adult-related trauma, such as intimate partner violence (IPV), has also been shown to compromise mother-infant bonding. Zeitlin and colleagues found that mothers experiencing IPV were less likely to bond well with their fetus, and as a result, were more likely to experience weakened mother-infant bonding compared to mothers without an abuse history [3]. Maternal depression seems to explain some of the associations observed between traumatic histories (e.g., early childhood abuse and IPV) and mother-infant bonding [4, 5].

Studies have also suggested that mothers with depressive symptoms are more likely to experience weakened mother-infant bonding. Muzik et al. observed that women with postpartum depressive symptoms experienced greater bonding impairment compared to non-depressed controls at 6 weeks, 4 months and 6 months postpartum [1]. Another study of 101 Caucasian women, who on average were 33 years old, demonstrated that maternal depressive symptoms at 2 weeks, 6 weeks, and 4 months postpartum were associated with lower quality of maternal-infant bonding [6].

To elucidate the relationship between traumatic events over the woman’s lifetime, maternal depression and mother-infant bonding, investigators have begun to explore the role of hormones, specifically oxytocin. Oxytocin (OT) is often colloquially referred to as the “love hormone” because it is involved in amorous engagement and maternal bonding. OT is a positive feedback hormone, synthesized in the hypothalamus and released from the posterior pituitary gland. It is produced during labor in response to contractions of the cervix and uterus, during physical bonding, and during breastfeeding (see [7] for review, [8]). Lower OT levels have been associated with impaired mother-infant bonding and maternal depressive symptoms [9–12]. In addition, others have described lower levels of oxytocin as a mediator between childhood and adult trauma and weakened bonding [13, 14]. A systematic review reported that 7 of 8 studies showed strong to significant associations between oxytocin levels or patterns and aspects of mother-infant bonding [7]. One study included in the review investigated the emergence of OT throughout pregnancy and postpartum. Results showed that rather than specific levels of OT being a significant indicator of bonding, a lack of overall increase in OT levels from early to late pregnancy correlated with lower quality maternal-fetal bonding and consequently lower quality maternal-infant bonding [9].

A recent review of the literature demonstrated that decreased OT levels were also associated with increased depressive symptoms [11]. One study examining the association between plasma OT levels and postpartum depression among 100 women in Switzerland found that lowered OT was a significant predictor for postpartum depressive symptoms [10]. Furthermore, another study revealed that lower OT was significantly associated with depressive symptoms in a sample of primarily Caucasian (83%) and African American (15%) women at 8 weeks postpartum [12].

A relationship between trauma and OT has also been demonstrated. Heim et al. observed lower concentrations of OT in a sample of 22 women who experienced childhood adversity, the majority of whom were Caucasian (68%) and African American (32%), suggesting a negative correlation between childhood maltreatment and OT levels in adulthood [13]. As for more recent adverse life events, a study by Samuel et al. examined the association between recent stressful life events and the prediction of OT levels in a sample of 30 Canadian women with mood and anxiety disorders [14]. The study demonstrated that in women with high levels of cumulative psychosocial adversity, those who had experienced a stressful life event within the last year had significantly lower levels of OT prenatally [14]. Given the associations between lower OT and impaired maternal bonding as well as postpartum depression and maternal adversity, it is important to further investigate these associations in Latinas given their high rates of adverse life events (e.g., poverty) [15, 16] and postpartum depression (PPD) [17, 18].

Despite advances in the field, much of the research has not included immigrant and US-born Latinas. This population is important because, as a group, they have high rates of fertility [19], traumatic events over their lifetime [20, 21], and PPD [17], all of which have been associated with lower OT. The objective of this exploratory study was to assess the association between traumatic life events in childhood and adulthood, PPD assessed at 4 and 8 weeks post-delivery, maternal bonding scores gathered at 4 and 8 weeks postpartum using the Postpartum Bonding Questionnaire (PBQ), and OT levels collected at 8 weeks postpartum in a sample of Latina mothers.

## Methods

### Procedures

The study was conducted in Chapel Hill, NC between July 2013 and August 2014. Recruitment was conducted from July 2013 to May 2014. Participants were screened for eligibility during routine prenatal visits at the University of North Carolina at Chapel Hill Women’s Hospital by trained bilingual (Spanish and English) female research assistants. Women were handed a recruitment flyer that listed key eligibility requirements, such as third trimester of pregnancy, age, Latina descent, and whether they were interested in participating in the study. Prospective participants then informed research assistants of their interest and whether they met the listed criteria (e.g., Latina descent). To be eligible to participate, women had to self-identify as Latina, 18 to 45 years of age, have a singleton pregnancy, be able to read, write, and speak English or Spanish, intend to breastfeed ≥ 2 months, and be willing to be followed until 8 weeks postpartum. Exclusion criteria included self-reported maternal or infant disorders that might interfere with breastfeeding, substance use, and current or past severe psychiatric disorder other than unipolar depression (e.g., bipolar disorder). Sixty-five self-identified prenatal Latinas were screened for eligibility and were contacted by phone to participate in the study. Of the 65 screened, 34 women were available and willing to participate. Thirty-one women screened who agreed to be contacted about the study were not enrolled: nine were not eligible (e.g., under age), seven reported transportation difficulties, 12 were never reached, and three refused.

Women enrolled into the study were assessed three times: 1) in person during their third trimester of pregnancy when written consent was secured, demographic data was collected, and a complete psychological assessment was conducted that included traumatic histories; 2) a phone interview was conducted at 4 weeks postpartum at which time depression, bonding, and breastfeeding status were collected; and 3) at 8 weeks postpartum women attended an in-person laboratory interview where depression, bonding, breastfeeding status, and biological data were gathered. The time points were modeled after a prospective study of maternal mood and hormone function [12]. The week 4 interview was conducted by phone to reduce participant burden. The study was approved by University of North Carolina at Chapel Hill (#17342) Institutional Review Board. All women gave written consent to participate in the study. Women were compensated for their time following each interview.

### Measures

Mother-infant bonding was assessed using the Postpartum Bonding Questionnaire (PBQ; [22]), a widely used measure designed to capture possible disorders in the relationship during the postpartum period. The PBQ consists of 25 items that make up four subscales: Impaired Bonding (12 items), Rejection and Anger (7 items), Anxiety about Care (4 items) and Risk of Abuse (2 items). Mothers rate each item on a 0–5 scale ranging from never to always, with specific items reverse coded. Responses are summed, with a higher score representing compromised bonding. The cutoff score for Impaired Bonding is 12, Rejection and Anger = 13, Anxiety about Care is 10, and Risk of Abuse = 3 [23]; this last subscale was excluded from the analysis because none of the participants endorsed any of the items. The measure has been validated with Spanish-speaking and Latina immigrant mothers [24, 25]. The scales used here have been shown to have acceptable Cronbach’s alphas levels: 0.86 for Impaired Bonding, 0.89 for Rejection and Anger, and 0.67 for Anxiety about Care [26]. This measure has been used with Latina mothers [25]. The PBQ was administered at 4 and 8 weeks postpartum.

Depression was determined using the Edinburgh Postnatal Depression Scale (EPDS; [27, 28]), a reliable 10-item measure of depression pre- and post-delivery [29]. Scores range from 0 to 30 and a cutoff score of EPDS ≥ 10 is used to determine the presence of minor and major depression [30]. This cutoff score has been used with perinatal Latinas [21, 31, 32]. The continuous score and depression cutoff (yes/no) were used in the analysis. Depression status was assessed at enrollment in the third trimester of pregnancy, as well as 4 and 8 weeks postpartum.

Two measures were used to determine traumatic events over the lifetime: the Structured Clinical Interview for the Diagnostic and Statistical Manual of Mental Disorders – 4th edition [33], which captures childhood and adult-related adversity (e.g., intimate partner violence) as well as traumatic events involving the infant (e.g., infant demise or premature birth) and a structured trauma interview based on Diagnostic and Statistical Manual of Mental Disorders – 5th edition type traumatic events (e.g., sexual abuse, physical abuse, etc.) [34]. Women were also asked about trauma histories by the Principal Investigator or a trained research team member during the enrollment interview and during the 8-weeks postpartum laboratory visit. Interviews were conducted in English or Spanish based on participant preference. Responses were coded as yes if the participant reported a childhood or adult traumatic event or no if none such events were endorsed, which provided a count for the number of childhood or adult-related events. For reporting purposes, all childhood-related traumatic events (e.g., sexual abuse) were grouped into one variable as were adult-related traumas (e.g., intimate partner violence). Any traumatic events involving the woman’s child were grouped together. To capture lifetime trauma, all forms of trauma were summed.

At 8 weeks postpartum, women completed a laboratory protocol that allowed for the collection of OT (see [35] for details). A trained nurse placed an intravenous catheter in the woman’s antecubital vein to collect blood for OT. Women chose to feed their infant either using bottle or breast for 10 min. Blood was drawn 10 min before the feeding session began, at minutes 3, 7 and 10 during feeding and 10 min after the session ended. The timing was based on prior research with postpartum women and the pulsatile nature of the hormone [12]. Blood samples were collected in pre-chilled vacutainer tubes, then transported for processing on the same floor as the feeding session. Samples were cold-centrifuged and aliquoted into pre-chilled cryotubes and stored in a refrigerator at the required − 80 °C. Enzyme immunoassay with extraction (Enzo Life Sciences, Farmingdale, NY) was used to assay the samples based on previous studies [12]. The sensitivity of the assay was 11.7 pg/ml with a standard range of 7.5 pg/ml to 1000 pg/ml; intra-assay variation was 4.8% and inter-assay variation was 8%. Area under the curve was calculated to capture the repeated measurement of OT. This allows for the capture of the pulsatile nature of the hormone and use one variable that represents the overall concentration of the hormone collected during the infant feeding episode [12, 36].

### Data analysis

The sample was characterized using frequencies, percentages and descriptive statistics. Next, Spearman's rank correlations were used to investigate associations between continuous scores (e.g., EPDS scores, PBQ subscale scores and number of traumatic events). Point-biserial correlations were conducted to assess the associations between a history of a lifetime traumatic events or involving an infant (dichotomous) and PBQ subscale scores (continuous). Wilcoxon signed-rank tests were conducted to test differences in subscale scores over time. Kruskal–Wallis one-way analysis of variance were used to examine differences in PBQ subscales score and OT AUC by maternal depression status and traumatic events. Finally, linear regressions were utilized to assess interaction effects of traumatic event and OT AUC on PBQ subscale scores. Given the exploratory nature of this study, we did not control for multiple testing or repeated measures. All statistical analyses were conducted using SPSS version 23.0 [37].

## Results

**Fig. 1:**
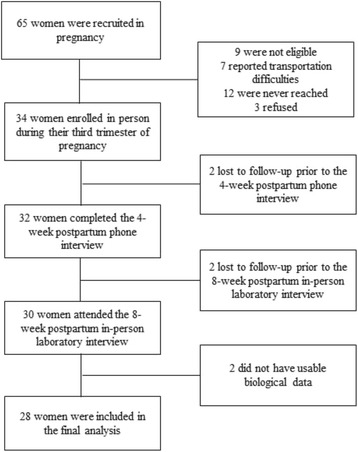
Subject flow chart

As Fig. 1 shows, 34 women were enrolled, 30 women completed the 4-week postpartum visit, and 30 attended the 8-week laboratory visit; four women were lost to follow-up. Of the 30 women who attended the laboratory visit, we were unable to establish an IV on two women. Thus, results are based on 28 women who had complete data from all three waves.

The final sample of women were primarily foreign-born (86%), married (82%), and not employed at the time of enrollment (79%). More than half (57%) had less than a high school education and were multiparous (64%). At 8 weeks postpartum, 82% of women were breastfeeding and 18% discontinued breastfeeding. During the laboratory visit, five women bottle-fed their infant, including two who had not stopped breastfeeding. Twenty-eight percent of women were depressed at enrollment, 18% were depressed at 4 weeks postpartum and 21% were depressed at 8 weeks postpartum. All mean PBQ subscale scores increased from 4 weeks to 8 weeks: Impaired bonding (*M* = 3.14, *SD* = 4.66) and (*M* = 4.93, *SD* = 3.71); Rejection and Anger (*M* = .68, *SD* = 1.79) and (*M* = 1.43, *SD* = 2.53); and Anxiety about Care (*M* = 1.25, *SD* = 2.07) and (*M* = 1.43, *SD* = 2.53), respectively. Results from the Wilcoxon signed-rank test indicated that there was a significant change in Impaired Bonding (*Z* = − 2.16, *p* = 0.031) from 4 to 8 weeks postpartum. Scores for Risk of Abuse subscale were not included in the analysis as all women scored zero at 4 and 8 weeks postpartum.

Sixty-eight percent of women reported any form of traumatic events. Half of all women reported at least one childhood traumatic event, 25% reported at least one form of adulthood trauma, 14% experienced at least one traumatic event involving an infant, and 18% reported more than one traumatic event over their lifetime. The most common childhood traumatic event reported was childhood physical abuse (25%), followed by sexual abuse (21%), and verbal abuse (14%); 14% experienced death of a parent and 7% reported parent abandonment. With regard to adulthood traumatic events, 25% reported physical abuse by an intimate partner, 18% experienced verbal abuse and 7% reported sexual assault. Fourteen percent experienced infant demise or a premature birth prior to enrolling in the study.

### Associations between maternal depression and mothers’ report of bonding

Prenatal depression scores were significantly correlated with Anxiety about Care at 8 weeks postpartum (*r* = .53, *p* = 0.004). Depression scores at 4 weeks postpartum were significantly correlated with Impaired Bonding (*r* = .42, *p* = 0.027) and Anxiety about Care (*r* = .50, *p* = 0.007) at 8 weeks. Depression scores at 8 weeks postpartum were also significantly correlated with Impaired Bonding (*r* = .56, *p* = 0.002) and Anxiety about Care (*r* = .59, *p* = 0.001) at 8 weeks. Significant differences in week 8 Anxiety about Care subscale scores by PPD status at 4 weeks were observed [H (1) = 6.78, *p* = 0.009].

Table 1 shows the distribution of PBQ subscales scores by PPD status at 4 and 8 weeks postpartum. Scores were higher among PPD mothers compared to non-PPD women. There was a significant difference by depression status at 8 weeks postpartum in Rejection and Anger subscale scores [H (1) = 3.72, *p* = 0.054], with higher mean scores among women with PPD (*M* = 3.33, *SD* = 4.32) than those without PPD (*M* = 0.91, *SD* = 1.57). No other significant differences were found.

**Table 1:** Distribution of postpartum bonding questionnaire subscales by postpartum week (*N* = 28)

	4 weeks	4 weeks	4 weeks	8 weeks	8 weeks	Week 8
	Depressed	Nondepressed	Total	Depressed	Nondepressed	Total
Variable	(*n* = 5)	(*n* = 23)	(*N* = 28)	(*n* = 6)	(*n* = 22)	(*N* = 28)
Impaired Bonding	6.80 (9.73)	2.35 (2.39)	3.14 (4.66)	8.50 (4.97)	3.95 (2.68)	4.93 (3.71)
Rejection and Anger	1.60 (3.56)	0.41 (1.16)	0.68 (1.79)	3.33 (4.32)*	0.91 (1.57)	1.43 (2.53)
Anxiety about Care	3.00 (3.74)	0.87 (1.36)	1.25 (2.07)	2.67 (2.39)	1.09 (1.57)	1.43 (183)

*Note.* Means and Standard Deviations shown

*p < 05

### Associations between maternal traumatic histories, PPD, and bonding subscale scores

Table 2 shows mean PBQ subscale scores by type of traumatic history. Women with histories of childhood traumatic events and infant-related traumas reported higher mean PBQ subscale scores at 4 and 8 weeks postpartum compared to women without those histories. However, women with at least one adulthood traumatic event (e.g., IPV) had lower mean subscale scores at both time points compared to those without such backgrounds. There were no significant correlations between the number of childhood traumatic events reported by mothers and any PBQ subscale scores or depression scores. There was a negative correlation between number of adulthood traumatic events and Rejection and Anger (*r* = −.41, *p* = 0.029) as well as Anxiety about Care (*r* = −.53, *p* = 0.004) at 8 weeks postpartum. Trauma history involving an infant was positively correlated with Impaired Bonding (*r* = .55, *p* = 0.003), Rejection and Anger (*r* = .60, *p* = 0.001), and Anxiety about Care (*r* = .65, *p* < .001) at 4 weeks postpartum, as well as Impaired Bonding at 8 weeks postpartum (*r* = .48, *p* = 0.009). Having a history of lifetime traumatic events was positively correlated with Impaired Bonding (*r* = .453, *p* = 0.015) and Rejection and Anger (*r* = .564, *p* = 0.002) at 4 weeks postpartum.

**Table 2:** Distribution of mean postpartum bonding questionnaire subscale scores by type of traumatic event (*N* = 28)

Variable	Childhood Traumatic Events		Adulthood Traumatic Events		Traumatic Events Involving an Infant		Lifetime Traumatic Events	
	Yes (*n* = 14)	No (*n* = 14)	Yes (*n* = 7)	No (*n* = 21)	Yes (*n* = 4)	No (*n* = 24)	Yes (*n* = 5)	No (*n* = 23)
Impaired Bonding								
4 weeks postpartum	3.71 (6.47)	2.57 (1.56)	1.43 (0.98)	3.71 (5.26)	9.25 (10.69)	2.12 (1.73)	7.60 (9.97)	2.17 (1.75)
8 weeks postpartum	5.71 (4.84)	4.14 (1.96)	4.86 (3.44)	4.95 (3.88)	9.25 (4.99)*	4.21 (3.02)	7.40 (5.98)	4.39 (2.95)
Rejection and Anger								
4 weeks postpartum	0.14 (0.54)	1.21 (2.39)	0.14 (0.38)	0.86 (2.03)	3.25 (3.95)	0.25 (0.61)	2.80 (3.56)*	0.22 (0.60)
8 weeks postpartum	2.07 (3.25)	0.79 (1.37)	0.14 (0.38)*	1.86 (2.80)	3.00 (5.35)	1.17 (1.81)	2.20 (4.92)	1.26 (1.82)
Anxiety about Care								
4 weeks postpartum	1.64 (2.37)	0.86 (1.70)	0.29 (0.76)	1.57 (2.27)	4.50 (3.42)*	0.71 (1.16)	2.80 (3.35)	0.91 (1.59)
8 weeks postpartum	1.71 (1.90)	1.14 (1.79)	0.00 (0.00)*	1.90 (1.90)	2.75 (2.50)	1.21 (1.67)	1.60 (2.61)	1.39 (1.70)

*Note.* Standard Deviations shown in parentheses

*p < .05

No significant differences in PBQ subscale scores were observed by childhood traumatic histories. Results from correlation analyses conducted on PBQ scores at 8 weeks were confirmed using Kruskal-Wallis. Significant differences in Rejection and Anger [H (1) = 4.12, *p* = 0.042] and Anxiety about Care [H (1) = 7.77, *p* = 0.005] by adulthood traumatic histories were observed at 8 weeks postpartum (see Table 2). Here, mean Rejection and Anger (*M* = 0.14, *SD* = 0.38) as well as Anxiety about Care (*M* = .00, *SD* = .00) subscale scores were lower among those with a history of adulthood traumatic histories than those without (*M* = 1.86, *SD* = 2.80 and *M* = 1.90, *SD* = 1.90, respectively). Differences in PBQ subscale scores by traumatic history involving an infant were also observed. There was a significant difference in Anxiety about Care scores at 4 weeks postpartum [H (1) = 5.13, *p* = 0.024] and Impaired Bonding at 8 weeks postpartum [H (1) = 4.20, *p* = 0.041], with higher mean scores among those who reported such a traumatic event. Results also revealed significant differences by lifetime traumatic histories in mean Rejection and Anger scores [H (1) = 5.88, *p* = 0.015] at 4 weeks postpartum; higher mean scores were observed among those with a history of lifetime traumatic events (*M* = 2.80, *SD* = 3.56) versus those without (*M* = 0.22, *SD* = 0.60).

### Oxytocin levels by PPD and trauma histories

Women with PPD at 8 weeks exhibited lower mean OT AUC (*M* = 688.87, *SD* = 168.04) compared to non-PPD women (*M* = 696.42, *SD* = 157.09), though the difference was not statistically significant. Table 3 shows mean OT AUC levels by type of traumatic events. Women with any form of traumatic history exhibited lower mean OT AUC compared to women without such histories. Results from the Spearman’s rank correlation analysis showed a significant and negative association between OT AUC and number of childhood traumas (*r* = −.40, *p* = 0.035); no correlations by bonding scores were observed. To further explore associations between oxytocin levels, PBQ subscale scores and trauma histories, we fitted several models and only those that reached significance are reported here. Results from the Kruskal-Wallis tests showed a statistically significant difference in OT AUC by childhood sexual abuse with [H (1) = 4.29, *p* = 0.038], with higher mean OT AUC (*M* = 724.12, *SD* = 157.23) among those without a history of childhood sexual abuse and lower mean OT AUC (*M* = 587.33, *SD* = 102.32) for those with such a history. Regression analysis revealed an interaction of AUC and childhood sexual abuse on Impaired Bonding (B = −.03, *p* = 0.038). No other significant associations were found.

**Table 3:** Mean and standard deviations for Oxytocin (OT) Area under the Curve (AUC) by type of traumatic event (*N* = 28)

Variable	OT AUC pg/ml
	*Mean* (*SD*)
Childhood Traumatic Events	
Yes (*n* = 14)	644.52 (129.13)
No (*n* = 14)	745.09 (169.19)
Adulthood Traumatic Events	
Yes (*n* = 7)	662.58 (126.86)
No (*n* = 21)	705.55 (166.35)
Infant-related Traumatic Events	
Yes (*n* = 4)	659.71 (126.64)
No (*n* = 24)	700.65 (162.30)
Lifetime Traumatic Events	
Yes (*n* = 5)	591.41 (70.82)
No (*n* = 23)	717.28 (161.61)

## Discussion

Results from this exploratory study revealed an interesting pattern by traumatic event type. We found that it is important to explore types of traumatic life events to unravel associations with depression, bonding and hormone function. Our analysis revealed that a history of adulthood traumatic events was associated with lower mean PBQ scores; higher scores signify compromised bonding. Contrary to previous findings (e.g., Kita et al., [4]) we might be observing a form of compensation among women who experience, say, IPV. In other words, these women might feel more protective of their infant and be especially aware of the care or protection their infant may need than other women in our study. Because we did not capture the proximity of the intimate partner violence to the timing the bonding subscales were administered, it is impossible to determine whether timing of the traumatic event influenced responses. Therefore, future investigations should capture the timing of the intimate partner traumatic event and completion of the bonding instrument to better estimate the effect of the traumatic event.

Our study also showed an interesting pattern as it relates to associations between a traumatic event involving an infant and mother-infant bonding subscale scores. We found that women who experienced such a traumatic event had higher mean Anxiety about Care subscale scores in the early postpartum period (i.e., 4 weeks). This form of traumatic event appears to have a long reach as women with such a history had higher mean Impaired Bonding subscale scores at 8 weeks postpartum. Given the fear and anxiety about the possibility of losing another infant, it is reasonable to expect that these women would have higher scores related to care of their infant. It will be important to understand how this early anxiety may translate into Impaired Bonding at 8 weeks postpartum. Further exploration of the potential effects specific or grouped items from the PBQ Anxiety about Care subscale have on infant care may shed some light. For instance, this subscale includes items regarding the woman’s confidence to care for her infant. This fear may translate into feeling detached from her infant—items that are included in the Impaired Bonding subscale. Therefore, it will be important to conduct a more nuanced examination to determine how specific items may help us understand mother-infant bonding scores, particularly among women who have suffered infant loss. It will also be important to follow women beyond the early postpartum period, as others have done, to help us better understand the evolution of mother-infant bonding in the context of trauma histories involving an infant [38, 39].

Depression during the early postpartum period (i.e., first 8 weeks) is also an important factor to consider when exploring mother-infant bonding and traumatic events among Latinas because of their high rates of PPD. In this study, close to a third of all women were depressed at enrollment, 18% were depressed at 4 weeks postpartum, and 21% were depressed at 8 weeks postpartum. These rates surpass those of the general population, who experience PPD between 10 and 19% [40]. In addition to finding significant correlations, our robust models showed that women suffering with PPD at 8 weeks postpartum had higher mean Rejection and Anger scores compared to non-PPD women. Previous studies have demonstrated similar associations, suggesting that higher levels of postpartum depressive symptoms may be predictive of women experiencing more feelings of rejection and anger towards their infant [41]. Lower socioeconomic status and a history of adverse life events are risk factors for PPD [42], and some research suggests migrant status also increases risk when combined with these other predictors [43]. Given the rates of such risk factors in our sample and Latinas in the US, it will be important to include a larger number of immigrant and US-born Latinas to examine the effect these risk factors along with PPD have on mother-infant bonding.

The secondary objective of this study was to explore the association between OT levels, an important biomarker in mother-infant bonding, depression, and traumatic histories in postpartum women. Our findings revealed that women with any form of traumatic event had lower mean OT levels compared to women without such histories. While these differences were not statistically significant, likely due to a lack of power, it shows the potential physiological effects of early and more recent traumas. This is consistent with other studies, which have found that women with traumatic childhood histories or those who have experienced recent stressful or traumatic events have decreased mean OT levels compared to those without [13, 14]. We also found that an exploration of specific traumatic events yielded interesting findings. Our analysis revealed that women with a history of sexual abuse exhibited significantly lower mean OT AUC levels versus women without. To our knowledge, this is the first study to demonstrate such differences. Given the potential lasting psychological effects sexual abuse can have on women’s mental health, these findings point to the importance of exploring the physiological effects such a traumatic event can have on a woman. This is especially important to explore in mothers, particularly given our finding that there was a significant interaction between a history of sexual abuse and OT AUC levels on Impaired Bonding subscale scores at 8 weeks postpartum. Again, we believe this is the first study to identify these associations. Given that oxytocin is involved in bonding and that sexual abuse histories have been shown to be associated with detrimental parenting behaviors, it is important to examine these relationships. Some studies have observed that mothers with histories of sexual abuse are more likely to demonstrate aggressive parenting behaviors, be permissive, emotionally withdrawn, or report more negative views about themselves as mothers [1]. It will be important to explore the mechanisms involved in higher mean Impaired Bonding in women with lower mean OT and sexual abuse histories.

While this study makes important contributions to the field, it is not without limitations. First, our study relied on a small, relatively homogeneous group of women. Consequently, we were not able to control for variables, such as education, parity and other demographic characteristics that might have affected the results. Therefore, the findings should be taken with caution. Future studies should include larger numbers of Latinas-both immigrant and US-born—to confirm the results reported here, explore associations that did not reach statistical significance here (e.g., differences in OT by trauma histories), and test possible mediations. Second, our study lacked control for confounding variables that might explain the associations reported here. Future studies should include larger samples of Latinas to allow investigators to test the effects confounding variables may have on the outcomes of interest. Third, given that this study only included Latinas, the generalizability of the results is limited. Therefore, future studies should include non-Latinas to increase the generalizability of findings. Fourth, we did not control for multiple testing and repeated measures on the same individual. This is particularly important given the potentially shared variance of the self-reported measures of trauma, depression, and bonding. To address these limitations, future studies should employ Bonferroni correction, and account for repeated observations as well as shared variance across self-reported measures. Finally, while there were no significant differences in OT AUC between women who bottle-fed and those who breastfed, future studies should capture mother-infant interactions during feeding observations to fully capture behaviors that might account for differences in hormone levels as well as maternal bonding. Subsequent studies that assess OT repeatedly over time should also consider using linear longitudinal modeling to account for those observations. Given the mixed effects exogenous oxytocin exposure (e.g., labor induction, labor augmentation, and/or postpartum hemorrhage prevention) has been shown to have on breastfeeding [44] and its potential long-term effect on endogenous levels [45], subsequent studies should measure exposure to account for potential differential levels by breastfeeding status and maternal-infant bonding.

## Conclusion

This first-of-its-kind study demonstrates the importance of exploring specific types of traumatic events to better understand associations between traumatic life events in mothers and infant bonding. The results also highlight the value biomarkers can add to furthering our understanding of factors associated with mother-infant bonding, particularly in mothers with a history of traumatic events.

## Abbreviations

AUC: Area under the curve
EPDS: Edinburgh Postnatal Depression Scale
OT: Oxytocin
PBQ: Postpartum Bonding Questionnaire
PPD: Postpartum depression
